# On Structure and Properties of Amorphous Materials

**DOI:** 10.3390/ma4091564

**Published:** 2011-09-15

**Authors:** Zbigniew H. Stachurski

**Affiliations:** Research School of Engineering, College of Engineering and Information Technology, Australian National University, Ellery Crescent, Acton ACT 2601, Australia; E-Mail: zbigniew.stachurski@anu.edu.au; Tel: +61-2-6125-5681; Fax: +61-2-6125-0506.

**Keywords:** classification of amorphous solids, atomic clusters, crystallisation, vitrification, glass transition, density fluctuations, ideal amorphous solid model

## Abstract

Mechanical, optical, magnetic and electronic properties of amorphous materials hold great promise towards current and emergent technologies. We distinguish at least four categories of amorphous (glassy) materials: (i) metallic; (ii) thin films; (iii) organic and inorganic thermoplastics; and (iv) amorphous permanent networks. Some fundamental questions about the atomic arrangements remain unresolved. This paper focuses on the models of atomic arrangements in amorphous materials. The earliest ideas of Bernal on the structure of liquids were followed by experiments and computer models for the packing of spheres. Modern approach is to carry out computer simulations with prediction that can be tested by experiments. A geometrical concept of an ideal amorphous solid is presented as a novel contribution to the understanding of atomic arrangements in amorphous solids.

## 1. Introduction

Crystalline solids have been known and appreciated since antiquity, in particular single crystal jewels. The elements of symmetry in single crystals of minerals were considered in modern times by R. J. Haüy in 1798. Soon after, the theoretical publications of W. H. Miller on *hkl* notation (1839), A. Bravais on 14 lattices (1845), A. Schönflies (1892) and W. Barlow (1898) on 230 space groups, with innumerable contributions from others, resulted in the modern theory of geometrical crystallography [1,2]. Theory of crystallography describes *ideal* crystal structures. The atomic arrangements of *real* materials depart in many ways from the ideal, hence the theories of crystal defects and disorder [3,4].

The discovery by René-Just Haüy [5] that a macroscopic crystal is made up of minuscule sized crystals all of the same shape, is the basis of crystallography. On extrapolation of this concept to the atomic level, the position of every atom in the perfect structure of any crystal can be predicted using the formula well-known to crystallographers:

(1) $${\vec{r}}_{atom}=M\left(r_{0},atom\right)+u\cdot \vec{a}+v\cdot \vec{b}+w\cdot \vec{c}$$

**Figure 1:**
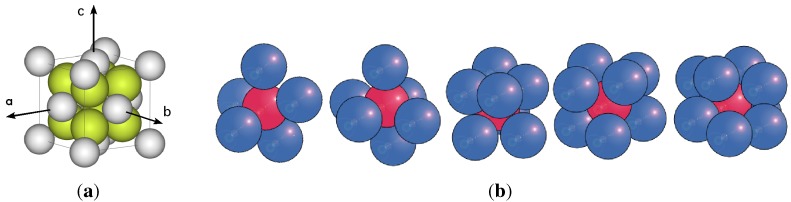
On left, an ordered crystalline motif of touching spheres. On right, random clusters with different number spheres touching the inner sphere.

Application of this formula with a given atomic motif (an example is shown in Figure 1a) creates a perfect crystal structure, usually represented by packing of spheres. A deduced geometric property of such a structure is that any straight line of arbitrary direction passing through the crystal will be divided by the atoms/spheres that it cuts through, or is tangent to, into finite segments recurring endlessly at regular intervals [6]. In the special case when such lines coincide with the directions of the vectors, $\vec{a}$, $\vec{b}$, or $\vec{c}$, the lines are referred to as the lattice. The knowledge of this property is used every time that X-ray diffraction is analysed, a single crystal jewel is cut, or the piezoelectric properties of a material are computed.

Considering now amorphous solids, it is conjectured that the positions of atoms cannot be predicted by any form of Equation (1), which implies translational symmetry. Instead, it is hypothesised that in a perfect amorphous solid any straight line of arbitrarily direction that passes through it will be divided by its spheres/atoms into *irregular* intervals of *random* sequence, which can be represented by a corresponding statistical distribution function.

In a historical perspective one can contemplate the following hypothetical question: If Pythagoras were a statistician rather than deducing perfect harmony from ratios of pure numbers, would we have had a theory of amorphousness developed ages ago? Both crystalline and amorphous solids were well known in antiquity. Leucippus and Democritus postulated atoms of different shapes, not related to the 5 ideal space filling solids. Yet, looking back in time one can draw a direct line from the modern theory of geometric crystallography to the philosophy of pure numbers and rational ratios of classical times. The Haüy description of packing of elementary blocks to form a single crystal with a simple relationship between its crystal faces and packing arrangement derives directly from the deductive Pythagorean notion of perfect harmony based on the relationship between the length of the string and perfect harmonic notes as 1:2, 2:3, 3:4, *etc*. The relationship between atomic planes and crystal lattice is also expressed by simple ratios of whole numbers, the reciprocals of which are the Miller indices. By comparison with crystallography, no such *universal laws or rules* are widely known about the structure of amorphous solids, consistent with the views expressed by Sheng *et al.* [7]:“…the atomic arrangements in amorphous alloys remain mysterious at present”.

Until quite recently, amorphous solids were considered to be disordered crystalline solids [8], thus originating from the corresponding crystalline state. One could say that to be disordered, there should exist a prior ordered state as a reference from which disorder can be measured. However, many amorphous solids do not have a corresponding crystalline form. Yet, it is still frequently assumed that amorphous materials are found at the limits of disorder, and amorphousness is usually defined by what it is not, rather than by what it is. A survey of experimental techniques will lead one to three methods for positively identifying amorphous materials: (i) calorimetry to measure glass transition temperature or associated thermodynamic quantities; (ii) X-ray scattering yielding the amorphous halo; and (iii) NMR to determine bond correlation. The last two methods essentially measure the same structural quantities.

**Figure 2:**
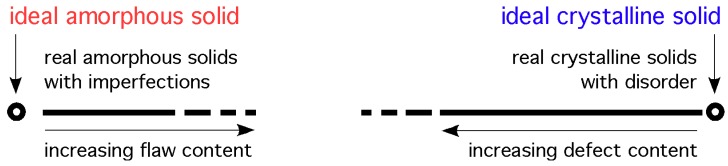
A view of the structure of solids along an undefined, somewhat arbitrary variable. The circles indicate the positions of the ideal (perfect) structures; the lines indicate the spectrum of structures in real solids.

In the field of geometric crystallography a disordered crystalline structure implies the presence of defects which are defined relative to the perfectly ordered structure [3]. Therefore, disordered materials are crystalline materials that can be, in principle, restored to the perfect crystalline state by the removal of defects. It is conjectured that this cannot be done for amorphous materials and that a different approach and terminology should be used to describe random atomic structure. To emphasise this point we note that in the field of statistics, when describing a set of *random* data, it would be unfitting to refer to that set as *disordered* data. Hence, it is proposed that amorphous structures, based on irregular packing of spheres, should be referred to as having a random arrangement of atoms rather than a disordered atomic arrangement. To promote this view a drawing is shown in Figure 2 with the envisaged position of the two types of solids, and the assumed discontinuity (however small) between the random and ordered types of atomic arrangements in solids. The small gap between the circle and the line on the crystalline side indicates that near-perfect single crystals can be grown. The larger gap on the amorphous side indicates that the structure of glasses may not be close to the ideal amorphous solid. A discontinuity in the line near the middle is meant to indicate that even highly disordered crystalline solids are not the same as highly flawed amorphous glasses, and vice-versa. This view is very close to that expressed by Kazunobu Tanaka *et al.* in the introduction to their book on amorphous silicon [9].

Nevertheless, the nature and origin of the amorphous state continues to be a scientific challenge [10,11]. This is still largely true today, and for this reason we advance and promote the theoretical concept of an *ideal amorphous solid* as a solution to this enigma. We believe that the right approach to a definition of amorphousness is through an appropriate geometric and topological model of the ideal amorphous solid as described herein.

However, the usage of the word “disordered" appears in dictionaries to mean unpredictable, opposite to law and order. So, this seems to be also a matter of habit and semantics, rather than a question of pure logic. Nevertheless, an appropriate and consistent vocabulary conjures up a clear vision of the atomic arrangements and helps to define the field of science of amorphous solids, separately from the field of crystallography.

The study of atomic arrangements in amorphous solids was stimulated in the 1960s by theoretical work of Bernall on the structure of liquids [12], concurrent with experimental random packing of spheres by Scott [13,14]. In the last decade research into atomic arrangements in amorphous solids has separated into two main streams: (i) more refined and complex studies of packing of spheres and molecules [15]; and (ii) atomistic simulations by molecular dynamics, including *ab-initio* methods [16]. The understanding we gain from the two approaches are of different nature. In the latter approach a unique definition of an amorphous atomic structure cannot be achieved simply because in a thermodynamic system without strong self-assembly tendencies (or in which such tendencies are frustrated) every simulation, even repeated on the same system, must result in a different atomic arrangement (depending on the initial conditions and thermodynamic path followed). Also, modelling amorphous materials by these methods is equivalent to random packing of spheres with extreme cooling rates of the order of $10^{15}$ K/s. However, these methods are reasonably successful and appropriate to simulate the structure of real amorphous solids with atomic arrangements containing imperfections. Therefore, the significance of the words “the atomic arrangements in amorphous alloys remain mysterious at present” is in recognising that the ideal (perfect) amorphous structure has not been universally accepted yet, although it has been defined for a certain class of amorphous solids [17]. In the former method we follow the methodology by representing atoms by spheres and creating a new model of random arrangement, totally different to crystalline structures.

There is a large body of published works on computer simulations of dense packing of spheres, which is beyond the scope of this paper to review. The quintessential aspects of such models are the rules established for packing (addition of spheres). Although many methods have been invented to add spheres in a “random" way, some do not result in ideal random packings because of preferred directionality or effects of specific forces, or by implied notions of a lattice with resulting bias towards regular structures. Some of the earliest models with well defined rules for packing of spheres were published by Finney [18], Adams and Matheson [19], and Bennett [20], and for the creation of a random 3D network by Ordway [21]. In the model of Bennett spheres are added onto sites formed by underlying 3-adjacent spheres, a key feature also used for the creation of an ideal amorphous solid, described herein later. However, Bennett’s model starts with a regular tetrahedron, and consequently contains imperfections in the random packing in the form of regular simplexes. A critical evidence for these imperfections is the split in the second peak of the pair distribution function, described adequately by Clarke and Jónsson [22] and by Donev *et al.* [23]. The model of Ordway has been developed further into an elaborate network model by Speedy [24].

The literature on the general principles for packing of spheres, with or without lattices, is extensive. For example: by Stoyan *et al.* [25], Conway and Sloan [26], Zong [27], Torquato [15], and many other notable contributions. In this paper we outline a unique model of an ideal amorphous solid as described previously elsewhere [17]. To model solids one has to consider rigid arrangements of hard spheres, which naturally leads to the so-called close packing of spheres. Close packing of spheres refers here specifically to the requirement that all spheres are in fixed positions [28], but not necessarily requiring maximum packing density [29,30]. Random close packing of spheres should be distinguished from random placing of dispersed spheres in space, of which one example is the Poisson point process [15,31,32].

In considering amorphous structures we discount solids which are crystalline materials but disordered to such an extent that long-range atomic order becomes undetectable. This corresponds to the case when atomic arrangement is disrupted, say, by heavy particle bombardment leading to so-called “amorphous" structure [33], or by heavy mechanical working (deformation). In principle, these solids can be restored to crystalline order by a suitable annealing. There is an adequate understanding of the observed changes in diffuse X-ray scattering caused by disorder [34].

## 2. Cognate Groups of Amorphous Solids

There are naturally occurring amorphous materials, such as obsidian mineral (vitrified rocks) and solidified organic materials, such as amber. Man made inorganic glasses date back to ancient civilisations (Summeria, Egypt). More recent additions to the family of amorphous solids are synthetic polymers (1940s) and metallic glasses (1960s). All of amorphous solids (natural and synthetic) can be separated into two broad categories [35]:

- Inherently non-crystallisable (equable glasses)
- Crystallisable (metastable glasses)

The first category is represented by solids such as cross-linked polymers (*i.e.*, diglycidyl ether of bisphenol A epoxy with 4,4${}^{\prime }$-diaminodiphenyl sulphone hardener), or atactic organic polymers (*i.e.*, a-polystyrene). The second category includes metallic and metalloid glasses, some metal oxides and linear macromolecular solids (polymers) in which the atomic arrangement is random in the glass, but in which crystallinity can be developed by annealing. Both characteristics can be combined, as in semi-crystalline polymers. All of these solids display common amorphous characteristics, such as X-ray scattering patterns in the form of an amorphous halo and the presence of glass transition temperature.

From the point of view of chemistry, all of the known amorphous materials can be segregated into (at least) 4 cognate classes as listed below.

### 2.1. Metallic Glasses

A relatively new group of solids that solidify in amorphous atomic structures are metallic glasses of the metastable category. Examples of commercial metallic materials are the Vitreloy alloys [36].

Crystallisation is suppressed by alloying a number of elements with selectively chosen atomic radii. The term “metallic" symbolises (to a first approximation) non-directional centro-symmetric atomic bonding. It allows a degree of freedom in forming varied atomic cluster arrangements as compared to the covalent or ionic bonding in organic and inorganic glasses in which coordination between atoms is limited to a small range [7,37]. Such man-made metallic glasses are heterogeneous in composition on atomic scale [15]. X-ray or electron scattering patterns show broad peak (amorphous halo) usually attributed to amorphous solids [38]. The liquid-to-solid transition is reversible to some degree, although crystallisation will occur eventually if annealing continues above the glass transition temperature [39,40].

Rules for the formation of these glasses have been published by Inoue [41] and by Ma [39], and by Yavari [42].

### 2.2. Inorganic Glasses

The structure of these solids is characterised by directional bonds that form 2D/3D networks with an average coordination number in the range between 3 to 5 (the coordination can vary significantly between the supercooled liquid and glassy solid). The random network is achieved by disrupting the pattern of covalent bonding (for example, additions of soda and lime to silica), thus preventing the natural tendency of silica to crystallise. At high temperature large proportions of the bonds dissociate and the 3-dimensional network dissolves into a mixture of low molecular weight fragments, resulting in a relatively low viscosity liquid. On cooling the network re-forms with viscosity increasing approximately as (mass of network − critical mass)${}^{-n}$, where n>1. The liquid-to-glass transition is reversible and repeatable. Annealing above the glass transition temperature may lead to partial crystallisation.

Rules for the formation of these glasses have been published by Zachariasen [43] and Warren [44]. Most inorganic glasses of this type form so-called “strong glasses”, with a narrow distribution in coordination numbers in the glass [45].

Amorphous ice (solidified water) apparently has several metastable forms [46], suggesting presence of polyamorphous glasses [42]. Amorphous silicon is a special glassy material. In one form it contains a significant hydrogen content, creating Si-H bonds. However, a chemically pure a-Si can also be produced (see for example reference [9]).

In this class we should also include amorphous carbon. The term amorphous carbon is restricted to the description of carbon materials with localised *π*-electrons as described by P.W.Anderson [47]. Deviations in the C-C distances greater than 5% occur in such materials, as well as deviations in the bond angles because of the presence of dangling bonds [48]. The above description of amorphous carbon is not applicable to carbon materials with two-dimensional structural elements present in all pyrolysis residues of carbon compounds as polyaromatic layers with a nearly ideal interatomic distance of a = 142 pm and an extension greater than 1000 pm.

### 2.3. Organic Glasses

These glasses naturally divide into three subclasses:

- random 3-dimensional (cross-linked) networks of copolymers,
- polymeric glasses based on atactic (non-crosslinked) macromolecules,
- polymeric glasses based on linear (iso- or syndio-tactic) macromolecules.

In the first subclass random networks form on copolymerisation of wide range of molecules with functionalities greater than 2, for example epoxy and with DDS as mentioned above [49,50]. The cross-linking reaction between the components takes place in the mixed liquid state (sol) which entrenches spatially random bonding resulting in a random, permanent, non-reversible molecular network. The initial liquid transforms into glass through the phenomenon of vitrification by increasing mass of the cross-linked network (gel) at the expense of the liquid (sol). Such a network can only be disrupted by thermal decomposition and oxidation at sufficiently high temperatures. Heating and annealing below decomposition temperature does not lead to “melting" or any molecular bond changes. No crystallisation can take place. These glasses do not become viscous liquids on heating above glass transition temperature.

The second subclass includes branched polymers with atactic architecture of the macromolecular chain (including random dendrimers). These macromolecular solids are restricted to form glasses on cooling from the molten state because the irregular chain structure prevents self-ordering and registering of the monomer units. The polymeric materials have permanent covalent bonds, unchanging between liquid and solid state. Annealing above the glass transition temperature does not lead to crystallisation. The liquid-to-glass transformation is, therefore, completely reversible and repeatable (providing oxidation is prevented).

The third subclass includes linear polymers that can be vitrified by quenching. In that sense their behaviour is similar to that of the metallic glasses in that on annealing, these glasses will develop crystallinity.

The rules for formation of amorphous polymeric solids were described by Angell [45] and Flory [49].

### 2.4. Amorphous Thin Films

Materials produced by methods akin to molecular vapour deposition techniques can form amorphous layers. Usually no specific information is known about the arrangements of atoms in these films; their amorphous nature is declared on the basis of absence of any crystalline X-ray diffraction peaks. (It is not generally known if they exhibit glass transition temperature). On heating, the amorphous structure rearranges and the transformation is non-reversible.

Low angle X-ray scattering from thin films has been reported by Rigden *et al.* [51], and the structure and properties of amorphous thin films has been described by Fuxi Gan [52].

Thin layers between crystalline grains may also exist in amorphous state, as described by Gleiter [53]. In fact, the original concept in physical metallurgy of “amorphous cement” at the grain boundaries was proposed nearly a century ago by Rosenhein, as recounted in the book by Cahn [54].

## 3. Density Fluctuations, Transport of Atoms, and Solidification

From the phenomenological point of view, solidification is the conversion of a liquid into a solid, where solid is defined as a body with memory of its shape of sufficiently long duration. A liquid can be transformed into a solid by cooling it. A liquid can also be transformed into solid by a chemical cross-linking reaction, or by applying hydrostatic pressure, which will not be considered here.

**Figure 3:**
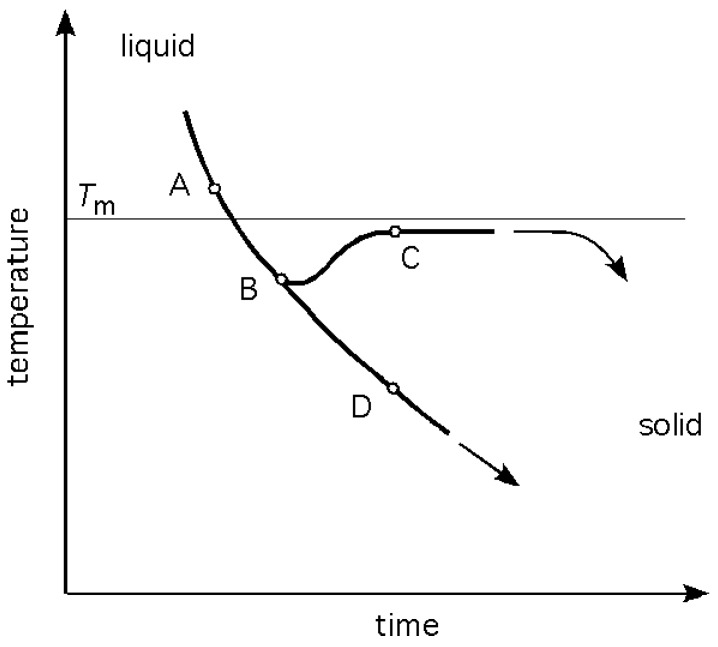
A portion of a cooling curve of a system undergoing solidification. $T_{m}$ is the equilibrium melting temperature. If nucleation and crystal growth occur the path follows A-B-C; if crystallisation does not occur, the path will continue along A-B-D.

As heat is taken away out of the liquid, at some rate $\dot{q}=\Delta q/\Delta t$, the temperature drops correspondingly at a rate, $\dot{T}=\dot{q}/mc_{p}$. The cooling curve follows a general exponential decay as shown in Figure 3. The bifurcation of the curve at point B indicates two possible paths below $T_{m}$, one resulting in a polycrystalline solid, and the other in an amorphous (glassy) solid. In either of the two cases the physical result is, after sufficient time is allowed for, a transformation of the liquid into a solid, albeit of different atomic structures. The word *bifurcation* is used to suggest that a change made to the system parameter values causes a ‘qualitative’ or topological change in its behaviour.

### 3.1. Atomistic Mechanics of Liquids

**Figure 4:**
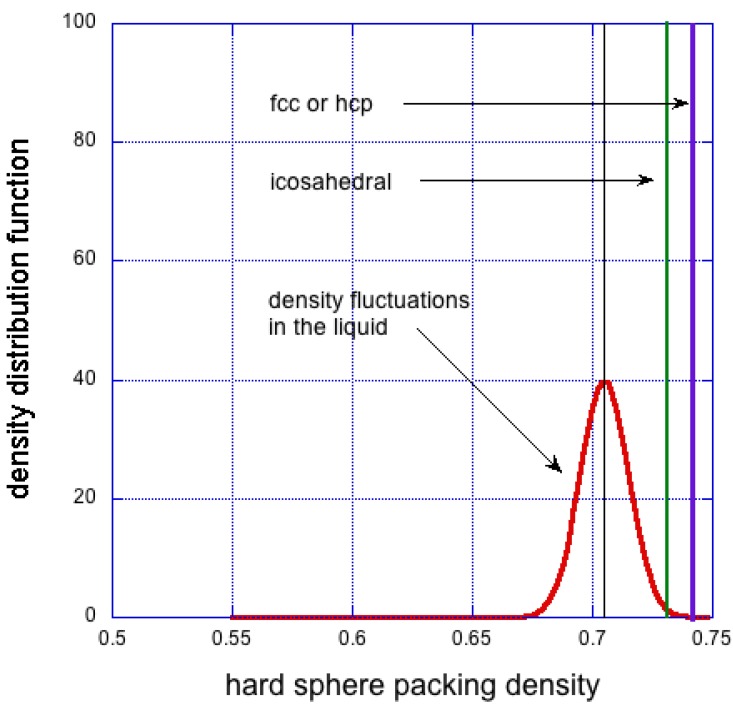
Expected density fluctuations in liquid phase (red). Also shown is approximate density of icosahedral cluster (green) and singular density of fcc crystalline solid (blue). The average density of the liquid is chosen to be 5% less than that of the fcc solid.

Below a certain critical thermodynamic point, the evolution of quantum states in the system, described *ab initio* by the Hamiltonian equation,

(2) $$|\phi \left(t\right)\rangle =\exp\left(-\frac{iHt}{\hbar }\right)|\phi \left(0\right)\rangle$$

leads to close range interatomic interactions and self-assembly tendencies. The many body interactions and their fluctuations, as implied by Equation (2), lead to description of the dynamics of the system, governed by the fluctuation-dissipation theorem. The spontaneous density fluctuations follow a random Gaussian distribution [55,56,57], as shown in Figure 4. Therefore, the density of the liquid at any point, r, can be thought of as an average density, $\rho_{0}$, and a superimposed periodically varying density $\rho_{p}$, such that:

(3) $$\rho_{l}\left(r,T\right)=\rho_{0}\left(T\right)+\rho_{p}\left(r,T\right)$$

The fluctuations at the high end of density result in ceaseless formation and solvation of atomic clusters of density higher than that of the average ($\rho_{0}$). Depending on certain physical and chemical conditions, these clusters can grow in size to metamorphose into crystalline nuclei, or cannot grow and remain in random arrangement as density fluctuations.

**Solidification by means of crystallisation** is well understood and soundly established [58], described by both (i) continuum thermodynamics; and (ii) atomistic mechanics.

### 3.2. The Essence of Continuum Thermodynamics

Along the path A-B-C, as the temperature drops from $T_{A}$ to $T_{B}$ below the equilibrium melting temperature, $T_{m}$, the Gibbs free energy of a solid becomes less than that of the liquid, $G_{s}<G_{l}$, so that $\Delta G=\Delta H_{f}-T_{B}\Delta S_{f}$ provides the thermodynamic driving force for the transformation. The subscript, *f*, indicates the change in *H* and in *S* on fusion. At the temperature $T_{B}$, the nuclei grow in size by absorption of atoms onto their surfaces in accordance with appropriate self-assembly rules resulting in crystallographic order. This is an exothermic process. With sufficient number of nuclei growing per unit time to overcome the prevailing heat loss, the temperature of the system will increase spontaneously, as indicated in Figure 3 by the path B-C.

These clusters, sometimes referred to as *embryos*, become stable *nuclei* on reaching the critical size,

(4) $$r_{crit}\geq 2\gamma_{A}/g_{V}^{c}$$

Within the crystal-like cluster of volume *V*, $\gamma_{A}$ is the surface energy per unit area *A* of the interface between the solid cluster and the surrounding liquid, and $g_{V}^{c}$ is equal to the internal energy of interactions per unit volume. In the classical theory of crystallisation it is assumed that $g_{V}^{c}$ and $\gamma_{A}$ are constant for a given chemically pure system. The arrangement of atoms in these nuclei is assumed to be the same as the arrangement of atoms in the corresponding crystal phase of the solid. For pure copper close to the melting point, $\gamma_{A}=1.5$ [J/m${}^{2}$], and $g_{V}^{c}=2$ [MJ/m${}^{3}$] [59]. Equation (4) predicts $r_{crit}=1.5$ [*μ*m] —too large for spontaneous density fluctuations. After crystallisation the density distribution changes dramatically from a Gaussian for the liquid for to a single valued *δ*-function for the solid (see Figure 4). The thermodynamic force of enthalpy of fusion overcomes the entropy of disorder.

It has been noted before that the maximum packing fraction of maximally random packed hard spheres is approximately 0.64 [60] whereas packing fraction of fcc or hcp arrangement is 0.74; the difference is over 15%—in stark contrast to systems such as pure metals (gold, copper, nickel) in which the interatomic interactions are close to centro-symmetric. The liquid and solid densities at their melting points are very close. For example, pure copper at $T_{m}$ has liquid density of 7.992 [Mg/m${}^{3}$] and solid density 8.352 [Mg/m${}^{3}$], a change of only 4.51% [61]. But in other systems it is much higher; for instance, for polyethylene it is 21% [62].

To resolve this problem, Frank [63], and Bernall [64], proposed that metallic liquids close to the freezing point contain clusters with icosahedral arrangement, which have much higher local packing density. Such an arrangement offers an intermediate step between random atomic packing and fully crystalline packing. It has density approaching that of the crystal, internal interaction energy comparable to that of fcc structure (at least for simple atomic liquids [65]), yet surface energy more than an order of magnitude lower, thus allowing much smaller nuclei to be stable.

The presence of clusters with five-fold symmetry has been corroborated by experimental evidence in liquid lead by Reichert *et al*. [66], by observation of quasicrystalline structures in solids [40,67], and supported by computer simulations [68,69,70,71] and by many others. A macroscopic icosahedral single crystal has also been grown [72]. But the probability of occurrence of icosahedral clusters is small, perhaps a few percent, and that of an fcc cluster is practically zero, as seen in Figure 4. Mossa and Tarjus [65] observed that transformation from random to icosahedral clusters propagates inwards from outside. They note that this is at variance with the classical homogeneous nucleation theory.

The overall view, summarised by Spaepen [73], is that the structure of supercooled liquids includes clusters that can be best described by packing of tetrahedra. For example, an icosahedral cluster involves packing together of 20 tetrahedra (somewhat distorted) and the resulting arrangement maximises the local density (close to, but not quite equal to fcc or hcp). The clusters immediately undergo rapid atomic rearrangements towards a more ordered, crystalline structure. At the temperature $T_{B}$, so close to the melting point $T_{m}$, this is essentially a high temperature annealing environment.

**Figure 5:**
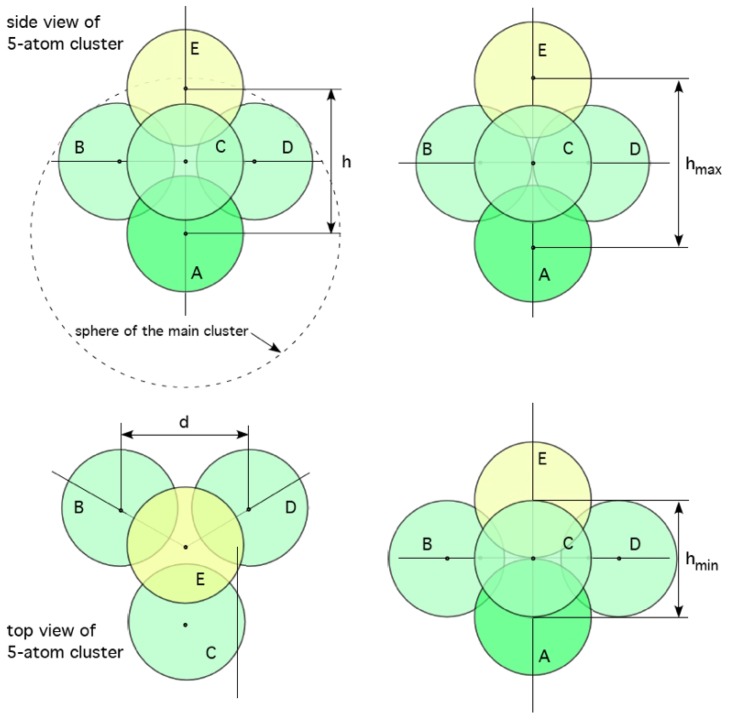
Models of atomic rearrangements in amorphous solids not requiring vacancy-atom exchange. Top, in the *α* mechanism atoms B, C & D move towards each other whilst A & E separate. Bottom, in the *β* mechanism atoms A & E come close at the expense of B, C & D moving further apart [74]. It is conjectured that these mechanisms supply additional pathways allowing for structural and chemical rearrangements within clusters and nuclei.

Diffusion rate within such a disordered cluster (nucleus) is rapid due to the high temperature and large number of defects. In addition to the classical vacancy-atom exchange mechanism for diffusion, there are *α* and *β* mechanisms for atomic rearrangements [74]. These involve relative movements of atoms in tetrahedral arrangements (characteristic of icosahedral packing) which lead to configurational and/or chemical composition changes requiring little free volume, as shown in Figure 5. A detailed atomic description of the mechanism has been described previously, in more general terms, as diffuse shear transformation by Argon [75]. The changes are driven by minimisation of free energy, but they also occur under superimposed mechanical stress, similar to the local rearrangements suggested by Mattern *et al.* [76], and for string-like cooperative motion suggested by Delogu [77].

**Figure 6:**
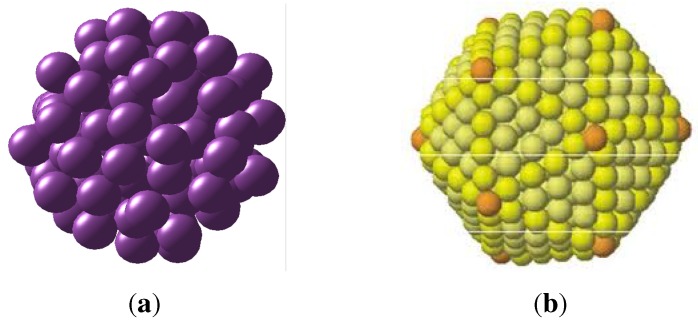
Models of monoatomic random clusters. Left, random cluster with 115 atoms with packing density of approximately 0.62. Right, an ordered cluster with icosahedral structure of 252 atoms and packing density of approximately 0.73.

These structural rearrangements by diffusion and other mechanisms will result in kinetically and thermodynamically stable nuclei with likely hybrid crystalline order, higher density and lower internal energy [78]. Examples of a random and an icosahedral cluster are shown in Figure 6.

In this representation of the solidification process there are three separately activated steps:

(1) formation of clusters of initially highly disordered/random atomic structure, where the rate of formation is given by: $\dot{N}=n^{*}\;\dot{\phi }$, with $n^{*}$ as the average number of clusters with critical size given by Equation (4), and $\dot{\phi }=\sum k_{a}\;\phi_{a}-\phi_{d}\sum k_{d}$ is the net arrival of atoms to the clusters with $k_{a}$ and $k_{d}$ as rate constants for addition and dissolution of atoms from the surface,
(2) rapid rearrangements of atoms towards regular crystalline order, driven by minimisation of elastic strain free energy, and controlled by diffusion rate: J=-DΔc,
(3) crystal growth, described by Johnson–Mehl–Avrami kinetic equation with implied functionalities on temperature and structure.

Step one removes the difficulty with the formation of highly improbable nuclei of perfect ordered structure. Step two allows for the formation of ordered nuclei (required for step three) by annealing and ordering of the random clusters. Step three leads to the first order thermodynamic transformation from liquid to solid at a constant crystallisation temperature.

**Theory of solidification through glass formation** is continuing to develop. Two approaches, that of (i) a thermodynamic theory of Gibbs–DiMarcio [79]; and an (ii) atomistic theory of free volume of Cohen and Grest [80], have endured over many decades and form the basis of our understanding of this process, albeit incomplete. It is worthwhile noting the essence of the Gibbs–DiMarcio theory, which assumes that thermodynamic equilibrium is achieved at all stages of cooling (at infinitesimally slow cooling rate), and that certain collections of molecules have equilibrium amorphous properties in all temperature ranges. They refer to the amorphous state as *non ordered* rather than disordered, a subtle but may be an important distinction. The theory postulates that under this condition glass formation is achieved with the following condition for configurational entropy:

(5) $$S_{c}=S^{liquid}-S^{glass}=0,\;\mathrm{or that it reaches its lowest possible value}$$

**Figure 7:**
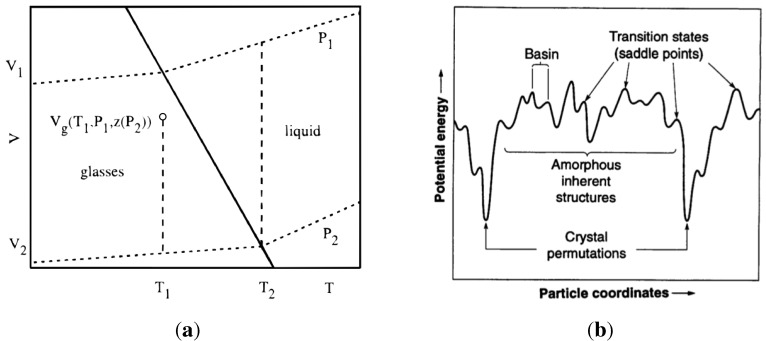
Volume-temperature phase diagram for a liquid-glass system. Reproduced from [81]. A schematic representation of the configurational phase space for glass. Reproduced from [82].

Their mean field calculation on the Flory–Huggins lattice model predicted a second order transition in the Ehrenfest sense, usually denoted, $T_{2}$, and the dependence of glass transition temperature on material properties: $dT_{g}/dP=\Delta \kappa /\Delta \alpha$. The derivation of this relationship assumed that volume, V(T,P) and enthalpy, H(T,P) of the system (at a given composition) are functions of the parameters, *T* and *P*, only. This is true for a liquid because its properties depend on *T* and *P* alone. As shown in Figure 7(a), $\left(V_{1}-V_{2}\right)$ is described completely by ${\left(\partial V_{1}/\partial P\right)}_{T}\left(T_{1}-T_{2}\right)$ and ${\left(\partial V_{1}/\partial T\right)}_{P}\left(P_{1}-P_{2}\right)$. For a glass this assumption is not valid, since the properties of glass also depend on its internal structure (atomic arrangements). Speedy [81] amended the Gibbs–DiMarcio argument by introducing an additional parameter for the glass, z(T,P), which describes the dependence of the glass structure on its volume, $V_{g}\left(T,P,z\right)$ and enthalpy, $H_{g}\left(T,P,z\right)$. Thus $\left(V_{1}-V_{2}\right)$ in the glass requires an additional term to close that path: ${\left(\partial V_{g}/\partial z\right)}_{T,P}\left(dz/dP\right)\left(P_{1}-P_{2}\right)$, as shown in Figure 7(a) by the gap between the end of the vertical dashed line at $T_{1}$ and the point $V_{1}\left(P_{1}\right)$. The dependence of glass transition on pressure now has an additional term that reflects the inherent structure of the glass:

(6) $$\frac{dT_{g}}{dP}=\frac{\Delta \kappa +\delta V_{z}/V}{\Delta \alpha }$$

In one of many papers, Stillinger [82] describes glass forming phenomena as indication of the multidimensional complex topography of the collective potential energy function, an example of which is shown in Figure 7(b). The system can explore the configuration surface among the higher lying amorphous inherent structures and attain a representative configuration, whilst avoiding crystal nucleation. A simulation study of the hard-sphere fluid shows that the statistical entropy, defined in terms of the configuration integral, decreases when it forms a glass, which indicates that a system of *N* hard spheres can form about exp(0.2N) glasses. For a system of N = 256 spheres studied there are about $10^{22}$ distinct glasses [83]. At high temperature the system continuously traverses over the potential energy landscape by means of thermal fluctuations. As the fluctuations decrease in amplitude (lowered temperature) the system may become arrested in one of the local minima. Which local minimum it will be trapped in depends on both, the amplitude of fluctuations and on being in the vicinity of the particular minimum. Every minimum (its depth) represents a particular atomic arrangement (structure) in the glass. The collection of all minima is called the *inherent structures* of the system. The behaviour of the system in the inherent structures, and transitions between the inherent structures, have been investigated by Tsalikis *et al.* [84]. They showed, with a detailed analysis, that above $T_{g}$ molecular dynamics simulation is capable of ergodic sampling of the potential energy landscape with relatively low computational costs. At $T_{g}$ and below, even with the best computational techniques employed, the virtual system becomes arrested where it found itself at the beginning of this range, for times far beyond the reach of molecular dynamics (MD) simulations. This is an important conclusion as it defines the limitations on MD simulations of amorphous structures.

The meaning of inherent structures may not be clear to a Materials Scientist with expertise in the measurement and description of atomic arrangements in solids, especially crystalline solids. As a first approximation towards understanding inherent structures, one can make an analogy between the dependence of glass structure (*i.e.*, atomic arrangement) on solidification conditions and that of the crystalline materials having their microstructure varying, depending on solidification conditions. Typical crystalline microstructures range from 1 μm grain size to that of a macro-sized single crystal, with large variations in crystalline defects and consequent properties. The aim of this paper is to give an account of the possible atomic arrangements in glasses, including the description of an ideal amorphous solid.

One of the salient points in Stillinger’s theory is the concept of an *ideal glass state* that could be experimentally attained if only sufficiently low cooling rates were available. This statement suggests that an ideal glass can only be obtained when the system has an infinite time to explore the *n*-dimensional potential energy space. It is, in fact, in accordance with the views of Gibbs–DiMarcio (Equation (5)). The ideal glass state achieves and equilibrium glass, which is not the same as the ideal amorphous solid.

**Figure 8:**
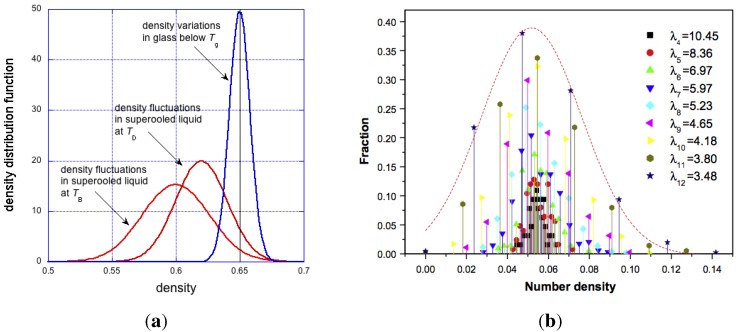
On the left: hypothetical density distribution in supercooled liquid at two different temperatures and in glass. On the right, density distribution in metallic glasses, reproduced from [70].

In the theory of Cohen and Grest [80] it is assumed that an amount of local volume is associated with each atom/molecule (atomic packing fraction). When it reaches some critical value, $v^{*}$, the excess is regarded as *free*. Atomic/molecular transport occurs only when $v^{*}\approx v_{m}$, the latter being atomic/molecular volume. Under this assumption, this can occur only in the low end of the density fluctuations shown in Figure 8a, where such holes/vacancies are likely to exist. As the temperature is lowered, the average density of the supercooled liquid increases, whilst the variance of the density fluctuations decreases as shown schematically in the diagram. This relationship continues below glass transition temperature, with one fundamental difference. The density variations in the glass are spatially frozen and time independent, whereas the density fluctuations in the liquid are stochastic in both time and space. In liquids and amorphous solids scattering of momentum is intermediate between (i) hydrodynamic behaviour where density fluctuation can be considered as a small perturbation in an isotropic continuum in thermal equilibrium; and (ii) at very short time and length scales, where dynamics can be viewed as that of independent particles between successive collisions.

A theoretical study of the distribution of free volume in hard sphere model liquid and glass has been carried out by Sastry *et al.* [85]. They defined *free volume* as the volume within which the centre of a given hard sphere can be moved without disturbing the position of neighbouring spheres, distinct from *cavity volume* as the space available for addition of another sphere. They have ascertained that the distribution of free volume in such a system is accurately described by the product of two factors: (i) $v_{f}^{a}$ with a≈0.3; and (ii) $\exp(-\beta v_{f}^{b})$ with b≈0.5 and *β* a temperature dependent factor. The peak of the distribution is around the volume fraction of 10${}^{-3}$, decreasing rapidly towards higher fractions. This means that free volume is essentially smoothly distributed over the system’s atoms.

An important inference, based on experimental facts, was made by Yavari *et al.*, that free volume in glasses is dispersed throughout the volume, but does not accumulate in holes or vacancies [86]. Free volume can be measured by X-ray diffraction methods, as well as by dilatometric measurements. When applied to glassy materials, X-ray and dilatation methods show that the average interatomic distance changes with temperature in the same way as the volume. However, it was established half a century ago that creation of vacancies (*i.e.*, atomic sized holes) in crystalline materials leads to volume changes, but no changes in interatomic distance (to a first approximation). This was corroborated by both X-ray diffraction and dilatometric measurements that distinguish between volumetric dilatation and interatomic distance. It re-affirms the view that free volume in glasses disperses throughout the volume, and atomic sized vacancies are unstable and therefore, non-existent. Furthermore, it emphasises the point that lattice sites are clearly identifiable in crystalline solids, but have no meaning in amorphous solids.

Positron annihilation spectroscopy results also reveal that atomic scale open-volume regions are reduced by annealing [87]. Starr *et al.* observed that the distribution of Voronoi cell volumes is not symmetrical [88], and that no significant correlation exists between local volume of a specific molecule and its mobility. This has been also noted by others [28,89].

However, if this were the case, then the Cohen–Grest condition, $v^{*}\approx v_{m}$, is never met, and therefore diffusion or creep would never occur. This difficulty is resolved in two ways. First, the *α* and *β* mechanisms of Figure 5 can operate when local $v_{f}<v_{m}$, and second, the dispersion of free volume is stochastic (see Figure 8) and the probability of voids in some regions of glass is, $P(v^{*}\approx v_{m})\approx 1$.

A number of theories of plastic deformation and diffusion in glassy materials are based on the assumption that free volume is concentrated in holes that could be identified as vacancies [75], and therefore this issue requires further evaluation.

The range and complexity of real amorphous materials and their behaviour has spurred on new theories for solidification (glass formation), for atomic arrangements in glass and for relaxation in the atomistic or mesoscale regimes. Some can be approximately described as topological or mesoscopic.

Microscopic density inhomogeneities are characteristic of real glasses, and define the volume relaxation time of the system. This was emphasised by Egami *et al.* [90]. In amorphous solids every site is different from others, due to the irregularity of packing, and also due to different sized atoms in multi-components glasses [88]. Density inhomogeneities in glass are not only related to the density fluctuations in liquid. It follows from the nature of random packing of atoms and congruency that clusters of high density must be surrounded by clusters of low density [17]. Random clusters of high density cannot grow in size with the same high density, unless short range order is developed, leading to nano-crystallisation.

The mode coupling theory [91,92] is an approach to the study of complex behaviour in the supercooled liquids, developed from the idea of a nonlinear feedback mechanism. It is possible to gain the impression that the densification occurs by gradual and uniform reduction in interatomic distances, in accordance with macroscopic thermal contraction of the volume.

**Figure 9:**
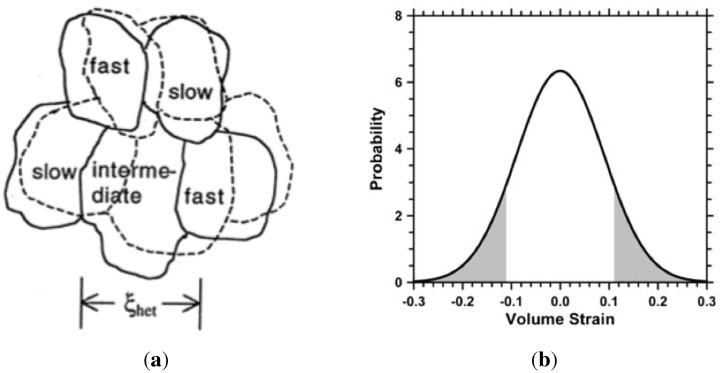
Dynamic inhomogeneities in density according to Ediger [56], and distribution of volumetric strain in random clusters according to Egami [93].

Ediger [56] considered the inhomogeneities of dynamics which in some regions (liquid-like) of the supercooled liquid can be orders of magnitude faster than dynamics in other regions (near-solid clusters) only nanometers away, as sketched in Figure 9a. This corroborates with density fluctuations, and is analogous with the formation of embryos and nuclei described earlier.

Egami has proposed a mechanism for the glass transition, based on topological changes resulting from variations in atomic level stresses [90,93]. Atomic sites with strain level greater than 11% are topologically unstable and will undergo atomic rearrangement. Sites with positive and negative stress levels will collapse and tend to annihilate the excessive strain levels. This is an improvement on the homogeneous free volume theory which fails to explain the relaxation of enthalpy and volume.

Returning to the issue of solidification, a system can follow the path A-B-D, leading to glass formation, under some specific physical and chemical conditions that disallow crystal formation. Latent heat evolution from this solidification process is negligible and insufficient to overcome the heat loss due to the applied cooling rate. Reducing temperature results in lowering of atomic mobility and diffusion rates in both liquid and solid phases, which in turn slows down atomic rearrangements. The atomic frictional forces follow the Vogel–Fulcher law: $\eta \propto \exp\left(A\cdot T_{V}/\left(T-T_{V}\right)\right)$, where: *A* and $T_{V}$ are constants characteristic of a particular system. As the volume fraction of high density inhomogeneities approaches a critical value, $\phi \to \phi_{0}$, the viscosity diverges rapidly [94].

Therefore, the favourable conditions for glass formation to occur are:

- when the formation of ordered nuclei is frustrated (as in multi-atom metallic alloys), thwarted (as in atactic polymers), or hindered (at extreme cooling rates),
- when the thermodynamic force (enthalpy) driving towards crystalline order is low and, therefore, the rate of ordering rearrangement is low (as in some binary alloys), then the clusters remain irregular and crystal growth does not occur.
- when covalent or ionic bonds form first (before solidification takes place) and bind the system into an irregular three-dimensional network (as in the case of network forming inorganic oxides or organic copolymerization),

As pointed out by To *et al.* in reference to an ideal amorphous solid [17], the density of glass can be increased by formation of imperfections in the random packing of atoms, or by reducing free volume and cavities. Densification can occur by the growth of the number of these clusters of high density, but not by growth in size of the clusters by surface addition of atoms. This is the fundamental difference from solidification by crystallisation. In summary, densification of the supercooled liquid and glass formation on cooling occurs by these mechanisms:

- reduction of amplitude of atomic oscillations (in both liquid and glass)
- reduction in free volume (in liquid)
- thermal contraction (vibrational anharmonicity, in glass)
- formation of imperfections with increased density (in glass)

It is conjectured that an incomplete understanding of glass formation is in part due to the lack of a coherent theory of amorphousness in which the central issue should be the definition of the atomic structure of *perfect*, defect-free amorphous solids. The knowledge of the atomic-scale structure of crystalline and pseudo-crystalline solids has been gained over the past century because of the developments in geometrical crystallography, of which the most important aspect is the concept of an *ideal crystal* in which unit cells form perfect ordered structures of infinite extent. Although real crystals only ever approximate this ideal (some not very closely), it is this ideal structure which is always used as a permanent base-line relative to which real crystalline materials are compared, and can be understood. The same methodology can be expected from a theory of amorphousness.

Perfection of structure may be defined firstly by the definition of imperfections that may occur, and secondly by the strict requirement of absence of those imperfections, namely no flaws or defects of any kind, for which we prefer the collective term *flaws* (in contrast to defects used in crystalline solids). For example, a gas in which the particle velocities conform precisely to the Maxwell distribution is referred to as an ideal gas; any deviation from this distribution is an imperfection. In the same sense, in an ideal amorphous solid the arrangement of spheres should conform precisely to distributions consistent with the coordination function Ψ(k), and the configuration function $P_{k}\left(\zeta \right)$, defined elsewhere [17]; any deviation is then an imperfection. Two general categories of flaws in the random amorphous structure can be defined: (i) geometrical; and (ii) statistical. Statistical flaws may have the effect of increasing the atomic packing fraction (density) of the structure. Specifically, clusters of 13 spheres with hcp or fcc arrangement are considered as serious flaws in the IAS, which increase drastically the average density of the body.

**Figure 10:**
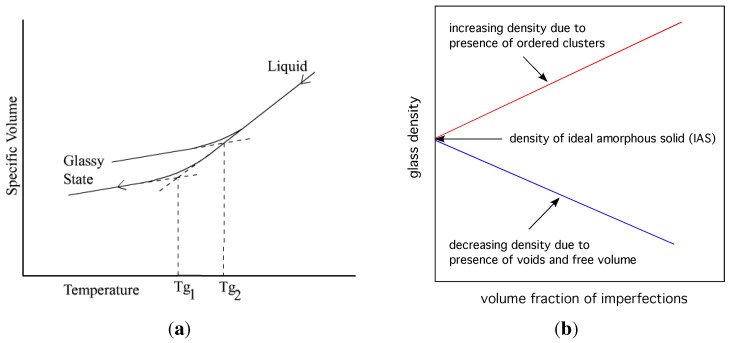
On the left, variation of volume with temperature for a glassy system; different glass transition temperatures for different content of imperfections. On the right, schematic representation of the effect that imperfections have on the density of glass.

Flaws undermine the structure of the ideal amorphous solid, causing its density to vary from the hypothetical ideal value. A presence of fcc, hcp and icosahedral clusters and similar cluster fragments (local crystallization) increases density, both locally and globally. A presence of free volume decreases density. It is conjectured from this viewpoint, that the viscosity of glasses and the glass transition temperature depend on the departure from ideal amorphous structure by the presence of imperfections in the real glass (see Figure 10). One can hypothesise that the glass transition should be separately related to these two factors. Some evidence for this, partially corroborating the hypothesis, has been published by Cheng *et al.* [69] who showed by simulations that icosahedral clusters in Cu${}_{64}$Zr${}_{36}$ alloy are of the slowest dynamics, and that there are more irregular liquid-like clusters in the high mobility regions.

**Figure 11:**
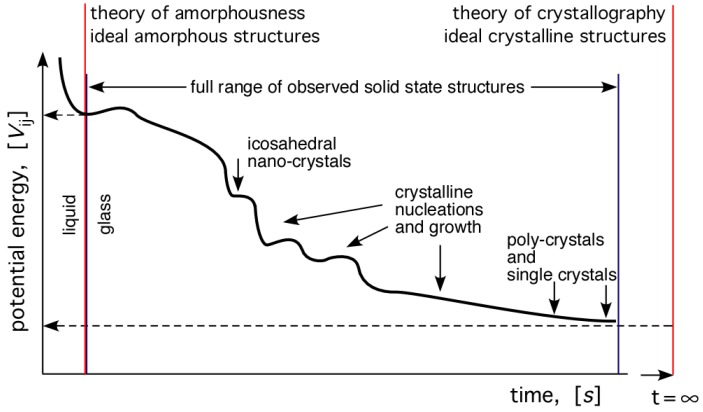
Schematic representation all possible atomic arrangement structures that may occur in a system cooled from liquid to solid. Note the two theories superimposed in the figure; the theory of crystallography, and a developing theory of amorphousness.

The continuation of the cooling process beyond the end of solidification, with all the ensuing possible phase transformations, can be illustrated schematically by the diagram shown in Figure 11. Naturally, this requires sufficient temperature and time frame to occur. The plot shows the variation of potential energy of the system as a function of time, decreasing with time until the asymptotic value for the single crystal structure is attained. The potential energy is in general given by summing all the interactions:

(7) $$V_{ij}=\sum_{i}v_{1}\left(r_{i}\right)+\sum_{i}\sum_{j>i}v_{2}\left(r_{i},r_{j}\right)+\sum_{i}\sum_{j>i}\sum_{k>j}v_{3}\left(r_{i},r_{j},r_{k}\right)+...$$

Indicated symbolically on the diagram are two theories of the geometrical structure of solids: (i) the theory of crystallography; and (ii) a newly emerging theory of amorphousness. Both describe idealised structures of atomic arrangements in the respective solids. Both are needed as the basis for understanding of the structure of solids. Theory of crystallography is placed appropriately at t=∞, whereas theory of amorphousness is placed (somewhat arbitrarily) at the liquid-solid boundary. Between these two limits, the full spectrum of all possible atomic arrangements in solids are included.

## 4. A Theoretical Model of Ideal Amorphous Solid

### 4.1. Imagine, Design, Create, Explore

A theory of amorphousness can be advanced on the basis of two axioms:

A1. In an amorphous solid any straight line of arbitrary direction passing through the solid will be divided by the atoms/spheres that it cuts through, or is tangent to, into irregular length segments, in contrast to crystalline solids where inter-atomic distances are regular.
A2. In any amorphous solid the statistics of the distribution of distances of the irregular line segments will be the same regardless of the chosen direction of the line. Consequently, the solid will be homogeneous and isotropic.

Structures, of any kind, are created by purposefully following carefully thought out rules of construction. The rules for creating a model of an ideal (theoretical) amorphous solid should be considered as analogous to the rules expressed by Equation (1) for creating a perfect (ideal) crystalline arrangement. However, the resultant amorphous structure must contain none of the elements of symmetry present in crystalline arrangements, such as mirror, rotation or glide, contained implicitly in Equation (1). Therefore, to create an amorphous packing of spheres, the rules must not enforce any ordering beyond the nearest neighbour, and therefore can apply only to adjacent spheres. Such basic rules for amorphous packing of spheres can be expressed as follows:

R1. Every sphere must be in *fixed* position so that the resultant structure is solid. A sphere is in fixed position when for 4≤k≤9 touching contacts with neighbouring spheres, no more than (k-1) are located on one hemisphere; for k>9 a sphere is always in a fixed position.
R2. Three adjacent non-touching spheres must form an *irregular* triangle. No two triangles have the same (identical) shape.

The first rule requires that all spheres in the amorphous packing must have touching contacts; the number of contacts for any individual sphere in a monoatomic structure is in the range of: 4≤k≤12. We call a set of spheres *a cluster*, comprising one sphere in the centre (inner sphere) and a number of outer spheres touching the inner sphere (also called shell or coordination spheres), as seen in Figure 1b. A consequence of this packing arrangement is that no four adjacent spheres (touching or not) will lie in a plane. The second rule implies that in each cluster there are adjacent but non touching spheres, with distributed distances between them limited to the range: $2r<d_{ij}<<2\sqrt{3}\;r$.

Rule 1 ensures that this packing of spheres models a perfectly rigid solid since no sphere in the body can be moved in any direction. Rule 2 ensures that none of the symmetry elements, mentioned above, will be present in the amorphous body. Beyond that, the rules do not stipulate any relationship between the spheres.

It transpires that there are no solutions for random packing of spheres in one or two dimensions under the above rules [95]. In three-dimensions, the ideal amorphous solid (IAS) proposed by To *et al.* [17] is a solution for a perfect random packing of spheres satisfying the rules and axioms listed above. The model fits into the ideal amorphous solid circle on the left in Figure 2. It represents the theoretical ideal state.

### 4.2. Construction of IAS

**Figure 12:**
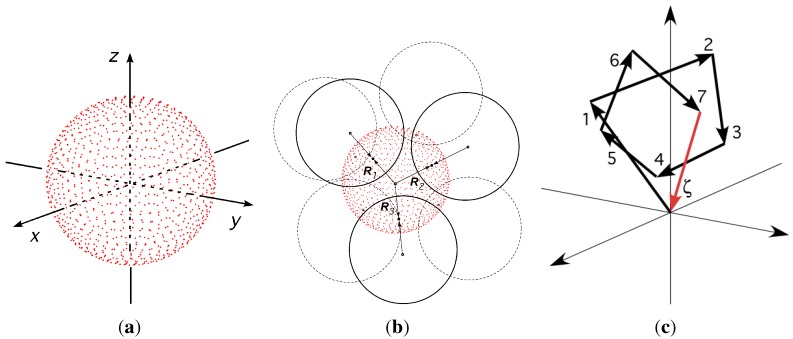
Formation of an irregular cluster as a seed for random packing of spheres by the method of To *et al.* [17]. On left, a sphere with $3.6\times 10^{3}$ equally spaced points. Centre, k=7 points selected at random and spheres added without overlap: $\pi /3\leq \phi (R_{i},R_{j})\leq \pi$. On right, vector polygon formed by radial vectors $R_{1},R_{2}$...,$R_{k},\zeta$.

**Figure 13:**
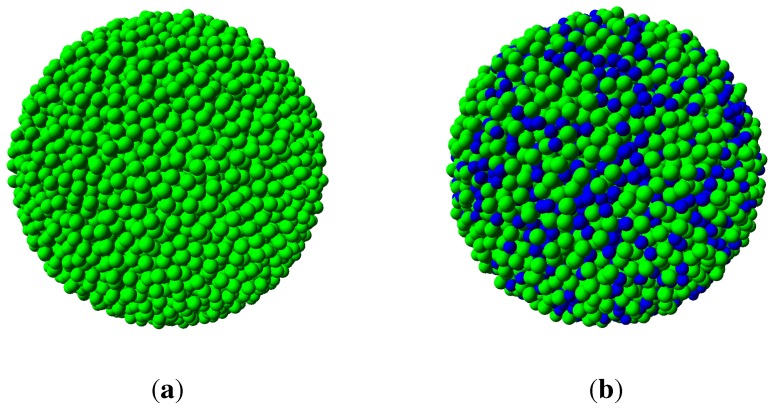
Monoatomic models of IAS. On left, a model comprising one type of spheres only. On right, a model with two types of spheres; blue spheres are 20% smaller than the green spheres. Each model contains 10${}^{4}$ spheres.

In practical terms, the construction of an IAS starts with placing one irregular cluster at the origin, created by first dividing the surface of the inner sphere into sufficiently many (say $10^{4})$ equally spaced points [see Figure 12a]. Next, *k* number of points are chosen *at random* (typically 7) as contact points for the coordination spheres. The points are ascribed radial vectors, $R_{i}$, that must have angular separation to avoid overlap of the outer spheres: $\pi /3\leq \phi (R_{i},R_{j})\leq \pi$. The cluster is completed by adding the chosen *k* spheres [see Figure 12b]. It should noted that infinitely many configurations are possible as the number of equally spaced points increases to infinity. In general, the added spheres do not touch each other (rule 2), but must touch the inner sphere below (rule 1). Next, new successive layers of spheres are added onto sites formed by 3-adjacent spheres below. The addition can be carried out, layer by layer, to the desired size of the amorphous structure (in theory, to infinity). Neither gravitation, friction nor vibration effects are applied during the construction. This process is described in detail in previous publications [17,74,96]; the computer code for simulation of the IAS is obtainable from http://www.materials.au. Examples of two IAS models created by this code, each comprising 10${}^{4}$ spheres, are shown in Figure 13a and Figure 13b.

### 4.3. Coordination in Clusters

**Figure 14:**
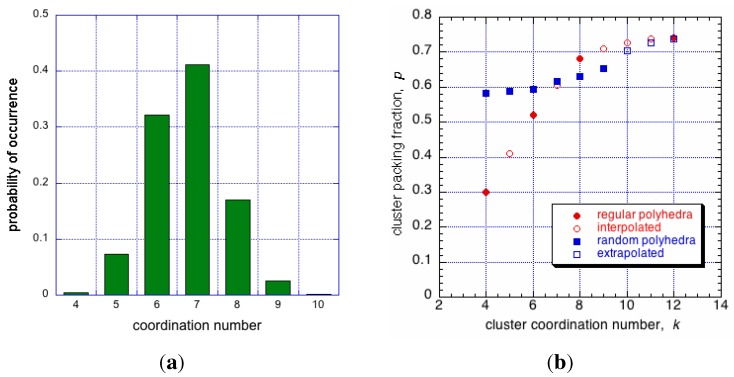
On left, probability of cluster coordination numbers in an IAS, based on Equation (8). On right, packing density of irregular clusters (in blue), and regular clusters (in red) as a function of coordination number, *k*. Monoatomic crystalline arrangement will have one coordination number only, an IAS will contain clusters with coordinations varying from 4≤k≤12.

Consequent on random packing is variation of coordinations in clusters, with admissible values for mono-disperse IAS in the range, 4≤k≤12. If c(k) is the fraction of clusters with a given contact number, *k*, then for the whole packed body of spheres, ∑c(k)=1, summed for k=4,…,12. Then the average number of contacts per sphere is: $\bar{k}=\sum k\;c(k)$, typically a non-integer, and possibly an irrational number. To a first approximation the occurrence of clusters with a given coordination number, *k*, is described by the combinatorics equation shown below, and plotted as the distribution function Ψ(k), shown in Figure 14a.

(8) Ψ(k)=xks-xx-k∕sx

where: *k* is the variable coefficient for the number of nearest-neighbour spheres relative to each inner sphere, x=12 is the maximum number of sites for spheres for close packing around the inner sphere, and s=21 is the number of virtual sites available to spheres in a less dense packing, as described in [17]. As seen in Figure 14a, the most frequent clusters have coordination number, k=7. The average packing density (or packing fraction) of clusters, as a function of *k*, is shown in Figure 14b by the blue points. Given the data shown in Figure 14, the average coordination number has been calculated as $\bar{k}=6.912$. An IAS body, such as show in Figure 13a, will contain the complete range of clusters, with consequent packing density variations that will be bounded by $\approx 0.59\leq \bar{pf}\leq 0.74$, a variation of approximately 11% from the mean. Interestingly, this shows that density-wise amorphous solids are less homogeneous at the atomic level than crystalline solids. In a crystalline solid every unit cell has identical composition, and every atom (of the same type) has the same coordination, and therefore the same packing density. But an amorphous solid has a distribution of clusters, each of different packing density (Figure 14b), and consequently density fluctuations extend for distances approximately an order of magnitude larger than that of a crystal unit cell.

### 4.4. Configuration of Clusters

The scalar quantity, *ζ*, defined by Equation (9), is chosen to be the random variable describing the configuration of clusters in the IAS. It is equal to the magnitude of the end-to-end vector, shown in Figure 12a, of the self-avoiding random walk formed by the radial vectors, $R_{j}$ for j=1 to $k_{\ell }$, for each *ℓ*-sphere, where *ℓ* is an index for spheres in the IAS:

(9) $$\zeta =|{\vec{\zeta }}_{\ell }|=\left|\sum_{j=1}^{k_{\ell }}R_{j}\right|$$

Let the probability density function of the random variable, *ζ*, be described by the equation:

(10) $$P_{k}\left(\zeta \right)=\left(4b^{3}/\pi^{1/2}\right)\zeta^{2}\exp(-b^{2}\zeta^{2})$$

**Figure 15:**
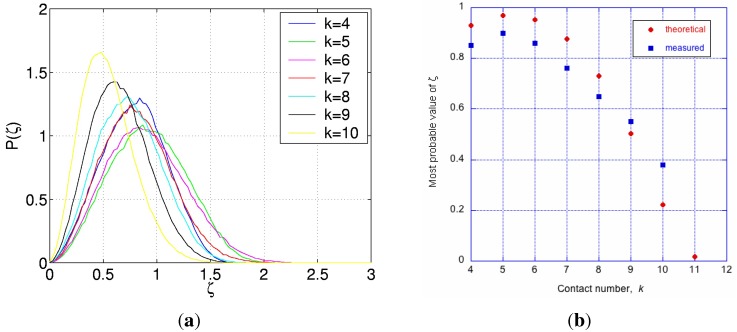
On left, probability functions for the random variable, *ζ*, for clusters with the values of *k* as shown. On right, the most probable value of $\zeta =\hat{\zeta }$ as a function of coordination number, *k*, predicted from Equation (11), and measured from simulations [17].

This function, $P_{k}\left(\zeta \right)$, is shown for selected values of *k* in Figure 15a. Clearly, for any cluster with a symmetric arrangement of its coordination spheres, ζ=0 and P(ζ)=0; also P(ζ)=0 for $\zeta \geq \zeta_{max}$, regardless of *k* [17]. Some similarity of this distribution to that published by Donev *et al.* [23] in their Figure 10, is incidental to the underlying statistical processes, however, the packing in their system is not perfectly random, and this may account for the rather symmetrical shape of that distribution described as Gaussian, unlike the case for the IAS shown in Figure 15a.

For a Gaussian random walk, the most probable value of $\zeta =\hat{\zeta }$ is proportional to $k^{1/2}$. However, as seen in Figure 15a and Figure 15b, $\hat{\zeta }$ varies with *k* in a way which is not in accordance with Gaussian random walk. This is because: (i) the consecutive steps on the radial vectors, $R_{j}$ for j=1 to $k_{\ell }$, are subject to hard sphere non-overlap condition leading to self-avoiding random walks (SARWs); and (ii) the SARWs are limited in extent by the finite space around the inner sphere’s surface. It is proposed that, as a first approximation, the most probable value, $\hat{\zeta }\left(k\right)$, of the self-avoiding space limited random walk can be described by the expression:

(11) $$\hat{\zeta }\left(k\right)=\left[\left(2R/3\right)\;k^{3/5}\right]\;\left[\exp\left(-\frac{\gamma }{1-k/k_{max}}\right)\right]$$

where: *k* is the number of steps in the random walk, $k_{max}=12$, and γ=0.4, is chosen to best fit the results. The exponent, 3/5, in first term is an approximate solution for SARW [28], and the second term represents the expectation of diminishing free space for the outer spheres as *k* increases towards $k_{max}$.

A comparison of $\hat{\zeta }$ predicted by Equation (11) with the values measured from computer simulations [17] is shown in Figure 15b. At least qualitatively, the trends are similar. However, Equation (11) is intuitive, and must not be taken as being empirical or validated.

### 4.5. Characterisation of IAS

**Figure 16:**
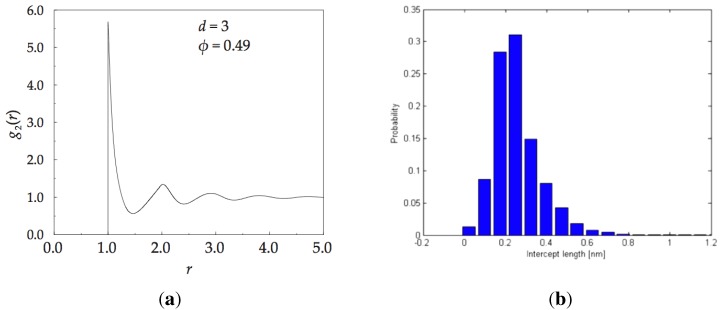
On left, pair distribution function for an monoatomic IAS. On right, statistics of the distribution of distances between atoms in a monoatomic ISA projected onto an arbitrarily chosen straight line regardless of the chosen direction of the line.

The statistical functions, $P_{k}\left(\zeta \right)$, and Ψ(k), characterise the randomness of the packing arrangement. Certainly, these functions provide a novel way to describe the structure of randomly packed spheres. Additional structural evidence is provided by: (i) pair distribution function; and (ii) X-ray scattering. A theoretically derived pair distribution function for the monoatomic amorphous solid has been calculated in reference [97], and it is shown in Figure 16a. It starts sharply at r=1, the closest approach of hard spheres. We have elaborated on these measures in previous publications for both, monoatomic and multi-atomic alloys [17,74,96].

The methodology of analysis is based on the fact that essentially all of the properties are related to each other through Huygens-style sum of amplitudes from all points of scattering and Fourier transforms. For instance; the local density at point, r, is related to the average density multiplied by the radial distribution function, $\bar{\rho }\times g\left(r\right)$, the radial distribution function can be derived from the X-ray scattering measurements, and the scattered intensity is computed from the Debye equation. All of these relations are well established and given in textbooks on structure of solids (for example reference [15]). An example of radial distribution function for the IAS is shown in Figure 16a, and the statistics of irregular intervals of any straight line of arbitrary direction passing through the solid, resulting from its division by the spheres that it cuts through, or is tangent to, is shown in Figure 16b.

That no short range order is possible in the IAS random packing of spheres can be reasoned as follows. Since all spheres are identical, variation in packing arrangement is the only source of differentiation. Then each cluster can be described by (at least) three statistics:

- the number of contact points, *k*
- the configuration of the nearest neighbours of the inner sphere, described by *ζ*
- the orientation of the cluster in space, described by some measure, Ω.

For two clusters (say, 1 and 2) to be identical it is necessary that [17]:

$$k_{1}=k_{2},\zeta_{1}=\zeta_{2},\mathrm{and}\;\;\Omega_{1}=\Omega_{2}$$

Each cluster has 9 possible values for k=4,…,12. If the distribution for *k* is equiprobable then the probability that two clusters have the same number of contact points (the same value of *k*) is simply 1/9. For any given distribution Ψ(k), as shown in Figure 14a, we can write more generally that the probability for two clusters to have the same value of *k* is given by $\sum_{k=4}^{12}{\left[\Psi_{k}\right]}^{2}=C/9$, where 1<C<9 is some known constant if $\Psi_{k}$ is known.

For the other two variables we note that two independent copies of random variables with continuous distributions agree with probability 0. Therefore, the probability that two or more neighbouring clusters have identical coordination and configuration, and are oriented in precisely the same way, is zero. Repeatability of an ordered or regular pattern in adjacent clusters extending along any line is zero. Consequently, it can be stated with generality that the IAS body has no short, medium nor long range translational and/or orientational order.

Such an ideal amorphous solid has no free volume, $v_{f}=0$, therefore, its configuration entropy is equal to zero, by definition. From this condition it can be deduced that such an ideal amorphous solid would possess Frenkel and Orowan theoretical strength, and that its coefficient of diffusivity would be equal to zero, D${}_{IAS}$ = 0. The same is true of a perfect single crystal as defined by crystallography.

### 4.6. Comparison with General Packings of Spheres

The packing of hard spheres is a long standing issue of topological research in space filling, both in terms of physical experiments, theoretical considerations, and by computer simulations. A search in library catalogues, or on the Internet, will reveal numerous publications in this field. So called ‘random packing of spheres’ is a special topic within this general area.

It is widely accepted, from an early paper by Scott and Kilgour on ’random’ packing of mono-sized ball bearings [14], that random close packing of identical spheres has a packing density close to 0.64. In a more recent publication by Aste *et al.* [98], and Yu *et al.* [99], densities close to 0.64 were also obtained, following gentle tapping or vibrations. X-ray tomography of the packing revealed the presence of clusters with ordered fcc and hcp arrangements. The crystalline fragments have formed by minute movements under the influence of vibrations, and self-assembly under the influence of gravity, and the same can be expected for Scott experiments. A simple first order analysis can show that the density of such a random close packing (rcp) will depend on the content of the “crystalline” fragments: $\rho_{rcp}=\left(1-\phi \right)\cdot \rho_{amor}+\phi \cdot \rho_{cryst}$ [17]; of special interest is the value of $\rho_{amor}$.

It appears that the value of 0.64 is also achieved in theoretical considerations [100] and computer simulations [101]. Torquato *et al.* introduced the concept of ‘maximally random jammed’ packing [29] with the packing density of ∼0.64. Another approach based on statistical mechanics predicts the maximum value of jammed random packing fraction as 0.634 [102]. Whilst the value of 0.64 appears to be ubiquitous, the precise structure of the packing is not specified uniquely, as different algorithms are used to create the random sphere packings, each unavoidably different. Without a formal definition of what a ‘random’ packing is, the issue remains unresolved.

Anikeenko and Medvedev reviewed shape characterisation methods for irregularity of the simplexes formed by sphere centres [100]. The *T* measure, given by $T=\left(1/15{\bar{l}}^{2}\right)\sum {\left(l_{i}-l_{j}\right)}^{2}$ summation for i<j, measures the irregularity of the tetrahedron formed by four adjacent spheres, in which $l_{i},l_{j}$ are the lengths of the edges, and $\bar{l}$ is the mean edge length. Clearly, for a regular tetrahedron, T=0. It is found that the maximally jammed random packings of spheres contain a fraction of tetrahedra with T=0, which manifests itself in the so-called split second peak in the radial distribution function [15]. However, as the fraction of these imperfections in random packing decreases, the evidence of their presence becomes statistically insignificant, and possibly overlooked. Nevertheless, they are *not perfect* random packings.

By contrast, the IAS described above and elsewhere [17,74,96] is based on a precise definition of random packing, embodied in the two axioms (A1 and A2), and the two construction rules (R1 and R2). The resulting structure is *ideal* in that it does not contain any packing imperfections (T≠0 for all simplexes and no split in the second pdf peak). The packing density of the mono-sized spheres IAS is found to be 0.625, although it must be verified by independent measurements.

It can be stated with generality, that the IAS body is homogeneous and isotropic down to a certain course-grain measure that can be specified by the mathematical statement:

(12) $$P(|\bar{\mu }-\bar{\rho }|\leq \tau )\geq \left(1-\alpha \right)$$

where: *P* stands for probability that the statement is true, $\bar{\mu }$ is the mean density of the region of interest, $\bar{\rho }$ is the mean density of the body, and the tolerance, *τ*, and the confidence limit, (1-α), are chosen in accordance with specific requirements.

### 4.7. Advantages of the IAS

(1) It is conjectured that the IAS is the only model of an imperfection-free, ideal amorphous solid for glassy metals with non-directional bonding.
(2) For amorphous, glassy materials, the IAS represents the base-line model for its atomic structure, and can be used to predict the *ideal*: (i) radial distribution function; (ii) structure factor; (iii) Debye X-ray scattering peaks; and (iv) coordination distribution; and (v) other topological properties.
(3) Specifying the atomic composition, *i.e.*, elements, concentration, and atomic radii, uniquely defines the ideal structure of the amorphous, glassy material.
(4) The IAS with 10${}^{5}$ atoms/spheres is simulated relatively quickly (minutes), and can be used as the starting structure for molecular dynamics.

## 5. Computer Models of Real Amorphous Metals and Alloys

Publications on computer simulations and structure of amorphous materials tend to contain mainly results of molecular dynamics simulations. Reviews of the methods have been published by Drabold [103], and in this issue by Valladares *et al.*[104]. In view of this, only a few selected papers will be mentioned with specific interests to the present review.

In computer simulations of molecular dynamics the hard, impenetrable spheres, are replaced by resilient atom-like objects with attractive and repulsive force-field around each. This change alters the packing geometry of the system, since the concept of an atomic radius, or a contact between two spheres is no longer valid. Consequently, an irreversible relaxation of the interatomic distances occurs during dynamic minimisation of system energy runs, which changes the coordination and configuration of each starting configuration of atoms. The most common starting configurations are based on packing of regular clusters without lattice, for example [86,105,106], sometimes employing two types of regular clusters [107]. On relaxation, the local atomic motions occur by the mechanisms described earlier (see Figure 5). This effect was also noted by Hui *et al.* [106] in their Figure 11, and by Liu *et al.* [108], who said that “adjustment of the relative atomic sites occurs without any long-distance diffusion".

**Figure 17:**
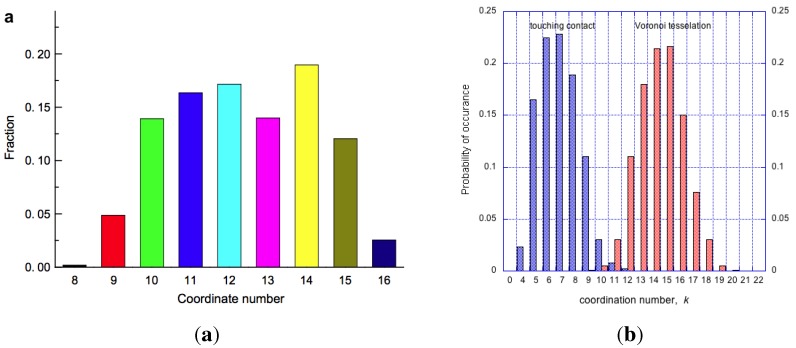
Distribution of coordination numbers in ZrTiCuNiBe metallic glass. On left, measured from molecular dynamics simulation by Hui *et.al* [106]. On right, coordination number distributions for the same alloy, but in an IAS model [74].

An interesting result on average coordination number distribution for multi-atom metallic glass has been published by Hui *et al.* [106], as shown in Figure 17. The different sizes of atoms present in the alloy allow for more efficient packing and a higher $k_{max}=16$ than that found for IAS. It should be noted that the coordination numbers for molecular dynamics simulations can only be obtained from Voronoi analysis, rather than touching contacts which cannot be defined in this case. For a theoretical IAS model of the same composition the coordination number distribution has been calculated, and it is shown for comparison on the right in the same figure. As expected, the static IAS model has a broader distribution, ranging from $k_{min}=10$ to $k_{max}=19$, consistent with the calculation showing that the largest diameter atom will have the highest coordination number [74]. Hui *et al.* make the point that different interpretations can be elicited from molecular dynamics and ab initio methods if no theoretical model is established first. The discrepancies between the distributions are ascribed to the packing imperfections that must exist in the molecular dynamics model, pre-supposing that the theoretical IAS has none. Such measures are beginning to evolve, and in due course they will allow a better understanding of the structure of amorphous materials.

**Figure 18:**
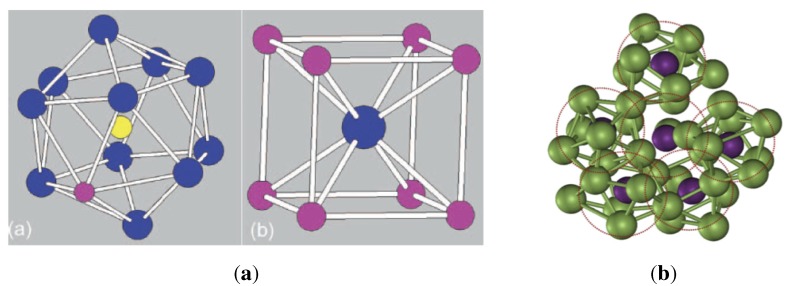
Another model of amorphous structure based on packing regular cubic and icosahedral clusters.

In the approach to modelling amorphous alloys by Fan *et al.* [107], it is proposed that two types of initial regular clusters are used: (i) an pseudo-icosahedral; and (ii) pseudo-body centred cubic, as shown in Figure 18. After molecular dynamics run with energy minimisation the resulting structure looses its regularity and assumes an amorphous structure, as shown on the right in the figure, albeit the structure is full of imperfections, or flaws, and subject to the uncertainly of the definition of cluster geometry. The appearance of the ubiquitous icosahedral cluster depends on the limits of distortion allowed [109].

The calculations of electronic states using density functional theory (DFT) are so advanced that structures and many properties of solids can be predicted with a good degree of accuracy. Software that couples the DFT and molecular dynamics (MD) simulation is now readily available. However, apparent success of such computations hide an inherent danger in the simulation of liquids and glasses, that is that the simulations are done at unrealistic time scales, and consequently some of the results could be incorrect [93].

## 6. Conclusions

1. The development of new bulk metallic alloys in the last 20 years, with extended supercooled liquid region, with high thermal stability and superior mechanical properties, has lead to resurgence of research activity in this field, both experimental and theoretical.

2. The field of amorphous materials, natural and synthetic, is extensive with widely varying chemistry and chemical bonding. Yet all of these materials display the same structural characteristics, of which the main ones are: (i) an amorphous halo in X-ray scattering; (ii) a glass transition temperature in thermal analysis; and (iii) electric, optical and mechanical isotropy.

3. A theoretical model of an ideal amorphous solid has been defined, paving the way for the development of a theory of amorphousness. Such a theory will provide: (i) a basis for characterisation of amorphousness of materials; (ii) a clear definition of amorphous structure on its own terms; and (iii) a definition of imperfections in the structure that are characteristic of real amorphous materials.

4. The development and application of density functional theory has allowed simulation of amorphous structures to a higher level of reality as compared to molecular dynamics computations. Computer software packages for simulation of amorphous structures are readily available to researchers. However, the size of the models (typically $<10^{4}$) is usually too small in most cases which does not allow for computations of bulk properties.

5. There cannot be permanency of site identification in amorphous alloys because of the non-existence of a “lattice", and the ease with which mechanisms *α* and *β* can change cluster composition.

6. As yet there are no sensitive techniques to measure the intricate details of the amorphous structure of materials. X-ray diffraction of crystalline solids gives sharp Bragg peaks capable of measuring interatomic distances and positions to a high accuracy. X-ray scattering in amorphous solids does not give the same high definition peaks, which at present limits the analysis. The same applies to calorimetric measurements. Other techniques may be capable of providing better resolution.

7. A theoretical ideal amorphous solid provides the base-line reference for metallic glasses; by definition it contains random packing and no imperfections.

8. Imperfections in amorphous solids can increase or decrease average body density, depending on the type and content of flaws present.
